# Insights into the role of RD3 in guanylate cyclase trafficking, photoreceptor degeneration, and Leber congenital amaurosis

**DOI:** 10.3389/fnmol.2014.00044

**Published:** 2014-05-26

**Authors:** Laurie L. Molday, Thomas Jefferies, Robert S. Molday

**Affiliations:** Department of Biochemistry and Molecular Biology, Center for Macular Research, University of British ColumbiaVancouver, BC, USA

**Keywords:** retinal degeneration 3, guanylate cyclase, photoreceptor cells, protein trafficking, photoreceptor degeneration, Leber congenital amaurosis, gene therapy

## Abstract

Retinal degeneration 3 (RD3) is an evolutionarily conserved 23 kDa protein expressed in rod and cone photoreceptor cells. Mutations in the gene encoding RD3 resulting in unstable non-functional C-terminal truncated proteins are responsible for early onset photoreceptor degeneration in Leber Congenital Amaurosis 12 patients, the *rd3* mice, and the *rcd2* collies. Recent studies have shown that RD3 interacts with guanylate cyclases GC1 and GC2 in retinal cell extracts and HEK293 cells co-expressing GC and RD3. This interaction inhibits GC catalytic activity and promotes the exit of GC1 and GC2 from the endoplasmic reticulum and their trafficking to photoreceptor outer segments. Adeno-associated viral vector delivery of the normal *RD3* gene to photoreceptors of the *rd3* mouse restores GC1 and GC2 expression and outer segment localization and leads to the long-term recovery of visual function and photoreceptor cell survival. This review focuses on the genetic and biochemical studies that have provided insight into the role of RD3 in photoreceptor function and survival.

## INTRODUCTION

Guanylate cyclase (GC) plays a key role in phototransduction by catalyzing the synthesis of cGMP. In the dark the basal catalytic activity of GC in photoreceptor outer segments (OS) is balanced by the basal activity of phosphodiesterase (PDE) to maintain cGMP at a level sufficient for maintaining a significant fraction of cGMP-gated channels in their open state. This allows the influx of Ca^2+^ and Na^+^ into the OS with the intracellular Ca^2+^ level reaching 250–500 nM (Gray-Keller and Detwiler, 1994; Woodruff et al., 2002). At this concentration, guanylate cyclase activating proteins (GCAPs) with bound Ca^2+^ maintain GC at its basal level (Gorczyca et al., 1994; Dizhoor et al., 1995; Polans et al., 1996). Following photoexcitation the Ca^2+^ level decreases to less than 25 nM through the combined closure of the cGMP-gated channels and continued efflux of Ca^2+^ by the Na/Ca-K exchanger (Woodruff et al., 2002). The reduced Ca^2+^ concentration triggers the exchange of Ca^2+^ for Mg^2+^ on GCAPs resulting in the activation GC (Peshenko and Dizhoor, 2006; Dizhoor et al., 2010). The increase in cGMP reopens the cGMP-gated channels and restores Ca^2+^ and cGMP to their dark state levels.

Vertebrate photoreceptors contain two membrane-bound GCs, generally called GC1 and GC2, that share a high degree of sequence identity (~49%) and structural organization (Shyjan et al., 1992; Lowe et al., 1995; Baehr et al., 2007; Karan et al., 2011). They consist of an N-terminal signal sequence followed by an extracellular domain, a transmembrane segment, a kinase homology domain, a dimerization domain, a cyclase catalytic domain, and a carboxyl-terminal extension. GC1 and GC2 exist as homodimers which localize to disk membranes of photoreceptor OS (Yang and Garbers, 1997; Ramamurthy et al., 2001). GC1 is present at relatively high concentrations in rod and cone OS, whereas GC2 is restricted to rod OS in mice and humans (Baehr et al., 2007; Azadi et al., 2010). The relative amounts of GC1 and GC2 vary with species with a GC1 to GC2 ratio of 4:1 in mouse (Peshenko et al., 2011b) and 30:1 in bovine photoreceptors (Helten et al., 2007; Kwok et al., 2008). The importance of GC1 and GC2 in the visual response and photoreceptor survival has been determined in knockout mice. In GC1 knockout mice, the cone photoresponse is absent and these cells undergo rapid degeneration (Baehr et al., 2007; Karan et al., 2010). Rod photoreceptors remain viable and functional due to the presence of GC2. In the GC1/GC2 double knockout mice, the rod and cone photoresponse is undetectable and both cell types undergo degeneration. The importance of GC1 in rod and cone function and survival is further highlighted by the finding that mutations in the *GUCY2D* gene encoding human GC1 cause Leber Congenital Amaurosis (LCA) Type 1 (LCA1), a severe early onset retinal dystrophy, and cone-rod dystrophy (Perrault et al., 1996; Kelsell et al., 1998; Karan et al., 2010).

Photoreceptor cells are highly polarized neurons with the OS segregated from the rest of the cell by a thin cilium. OS proteins must be efficiently transported from the endoplasmic reticulum (ER) in the inner segment through the cilium since OS turnover every 10 days through the phagocytosis of aged OS membranes by retinal pigment epithelial cells and the addition of new membrane at the base of the OS (Sung and Chuang, 2010). The molecular machinery which orchestrates the transport of proteins to the OS is highly complex involving vesicle budding, fusion, and motorized transport along microtubules and other cytoskeletal elements in the inner segment and cilium. Molecular-based studies indicate that the C-terminal targeting segment of several OS membrane proteins including rhodopsin and perpherin-2 play an essential role in vesicle trafficking through protein–protein interactions (Tam et al., 2000, 2004; Deretic et al., 2005; Chuang et al., 2007; Mazelova et al., 2009). The vesicle transport pathways, however, appear to be distinct for various OS membrane proteins (Fariss et al., 1997). Efforts to define a sequence required to transport GC to outer segments have been inconclusive (Karan et al., 2011). Recently, retinal degeneration 3 (RD3), a photoreceptor protein encoded by the gene associated with retinal degeneration in the *rd3* mouse, *rcd2* collie, and LCA12 patients has been shown to play a crucial role in the trafficking of GC1 and GC2 in photoreceptors (Azadi et al., 2010). In this review, we focus on the molecular properties of RD3 and its role in GC trafficking, catalytic activity, and photoreceptor degeneration.

## GENETIC AND PROTEIN ANALYSIS OF THE RD3

The gene responsible for retinal degeneration in the *rd3* mouse was first identified as an uncharacterized transcript in an *in silico* search of retina-specific transcripts and called *C1orf36* for Chromosome 1 open reading frame 36 (Lavorgna et al., 2003). The gene now known as *RD3* consists of 3 exons spanning the 5′ and 3′ untranslated region with the open reading frame comprised of part of exon 2 (amino acids 1–99) and exon 3 (100–195). RT-PCR and *in situ* hybridization confirmed the presence of *RD3* in the retina with high expression in the photoreceptors.

**FIGURE 1:**
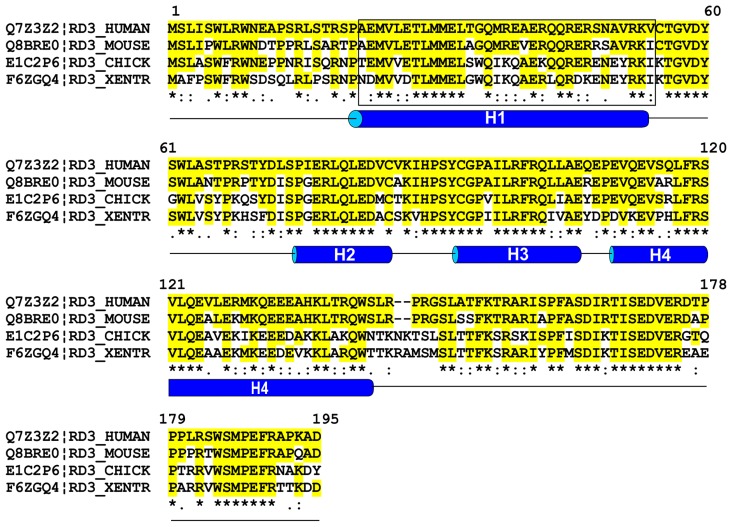
**Sequence alignment of RD3 proteins from various vertebrates.** Sequences of human, mouse, chick, and *Xenopus tropocalis* RD3 were aligned using Clustal W multiple alignment program. Sequence identities relative to human RD3 are shown in yellow and * and similarities are indicated by (:). Helical predictions were based on the PSIPRED protein structure prediction program and coil–coil domain (enclosed in a rectangle) was predicted using Coils program found in the web-based ExPASy Bioinformatics Resource portal.

The RD3 protein encoded by the *RD3* gene is highly conserved across vertebrates with the human protein consisting of 195 amino acids and sharing 95% sequence identity with other primates, 86% with mouse and rat, 83% with bovine, 67% with chicken, and 50–60% with lower vertebrates including *Danio rerio* (Zebrafish) and *Xenopus tropicalis* (Western clawed frog). Computer algorithms indicate that RD3 lacks any known homology domains or transmembrane segments. RD3 is predicted to have a high α-helix content (~43%), no β-sheets and considerable disordered regions (**Figure 1**). There are four conserved stretches of predicted α-helices (H1–H4) with the first helix consisting of 34 amino acids and the last helix having 39 amino acids. Additional conserved features include a putative coil–coil region between amino acids 22–54 and several predicted phosphorylation sites.

RD3 has been isolated from retinal homogenates and HEK293 cells and bacteria expressing the recombinant protein (Azadi et al., 2010; Peshenko et al., 2011a). On SDS gels RD3 migrates as a 23 kDa protein consistent with its amino acid sequence. Dynamic light scattering and gel filtration chromatography, however, indicate that purified RD3 in solution exists as a polydispersed oligomeric protein.

## TRUNCATION MUTATIONS IN RD3 CAUSE PHOTORECEPTOR DEGENERATION

The *rd3* mouse was one of the first naturally occurring murine strains found to display early onset rod and cone degeneration (Chang et al., 1993). The rate of degeneration varies with the background strain (Linberg et al., 2005; Friedman et al., 2006; Danciger et al., 2008; Molday et al., 2013). The albino RBF/DnJ strain shows the fastest rate of degeneration with only a monolayer of photoreceptor nuclei present 8 weeks after birth. The pigmented In(5) 30RK/J strain displays the slowest rate with some photoreceptors still present after 30 weeks. The Rb(11.13)4Bnr/J strain is intermediate in its rate of degeneration. Rod-derived scotopic electroretinograms (ERG) are measurable in all strains at an early age prior to significant photoreceptor degeneration (~24–35 days postnatal), but a cone response has only been reported in the In(5) 30RK/J strain (Friedman et al., 2006; Molday et al., 2013). The mutation which causes photoreceptor degeneration in all strains was first reported by Friedman et al. (2006). They identified a homozygous C→T substitution (c.319C→T) in the *Rd3* gene which causes a premature stop codon resulting in a protein lacking the last 99 amino acids.

An extensive human *RD3* mutational screen was carried out on individuals with autosomal dominant and recessive retinal degenerative diseases. A homozygous G → A transition in the donor splice site at the end of exon 2 was found in two siblings from a family with LCA (Friedman et al., 2006). This mutation causes a premature stop codon following codon 99 of the *RD3* gene. The *RD3* gene was the twelfth gene associated with LCA and accordingly this subclass is called LCA12. Subsequent genetic screens revealed homozygous mutations in *RD3* with severely truncated proteins in other LCA families (Preising et al., 2012; Perrault et al., 2013). World-wide genetic screens indicate that mutations in the *RD3* gene causing LCA are rare. In addition to truncation mutations, missense mutations in *RD3* have been observed in individuals with other retinopathies (Friedman et al., 2006). However, additional studies are needed to determine if these mutations are directly responsible for the disease.

A mutation in the canine *Rd3* gene causes rod-cone dysplasia 2 (*rcd2*) in a strain of collies (Kukekova et al., 2009). An insertion in the gene leads to an alteration in the last 61 codons and a further extension of the open reading frame.

Biochemical studies indicate that the truncated RD3 proteins associated with photoreceptor degeneration in the *rd3* mouse and LCA12 patients are highly unstable and non-functional (Friedman et al., 2006; Peshenko et al., 2011a). These studies support a crucial role of the C-terminal segment in the stability and function of RD3.

## INTERACTION OF RD3 WITH GC1 AND GC2

RD3 was first detected in a mass-spectrometry-based proteomic analysis of bovine photoreceptor OS (Kwok et al., 2008). Monoclonal and polyclonal antibodies against RD3 confirmed the presence of the 23 kDa RD3 protein in mouse retinal extracts by western blotting (Azadi et al., 2010). These antibodies, however, proved to be problematic for immunolocalizing RD3 in photoreceptors as they failed to consistently label retinal cryosections above background levels due to either the inaccessibility of the epitopes or the low level of RD3 expression. One polyclonal antibody showed some immunoreactivity in outer and inner segment layers above control retinas, but in subsequent studies this observation could not be reproduced (Azadi et al., 2010). Additional studies are needed to definitively determine the subcellular localization of RD3 in photoreceptor cells.

The first insight into the possible role of RD3 in photoreceptors was obtained from co-immunoprecipitation experiments. A monoclonal antibody to murine RD3 coupled to Sepharose immunoprecipitated GC1 and GC2 together with RD3 from mouse retinal extracts (Azadi et al., 2010). The interaction of RD3 and GC1 was confirmed in co-expression and co-immunoprecipitation studies. When RD3 and GC1 were co-expressed in HEK293 cells, an anti-RD3 antibody co-precipitated GC1 with RD3 and in a reverse experiment an anti-GC1 antibody co-immunoprecipitated RD3 with GC1 confirming the direct interaction between these proteins (Azadi et al., 2010). Analysis of GC1 deletion mutants further indicated that the C-terminus of GC1 is required for binding of RD3.

## RD3 IS IMPORTANT FOR THE EXPRESSION AND LOCALIZATION OF GUANYLATE CYCLASES

To determine the significance of the RD3-GC interaction, the expression and localization of GC1 and GC2 in the *rd3* mouse was compared with age-matched wild-type (WT) mice by western blotting and confocal microscopy (Azadi et al., 2010). GC1 and GC2 from WT mouse retina migrated as 120 and 125 kDa proteins on SDS gels, but were absent or present in reduced amounts in extracts from *rd3* mice depending on the strain used for the analysis (Azadi et al., 2010; Molday et al., 2013). At a subcellular level, GC1 and GC2 was localized to the photoreceptor OS of WT mice with GC1 present in rod and cones and GC2 restricted to rod cells as reported previously (Karan et al., 2010). In contrast, GC1 and GC2 immunolabeling was absent in 21-day old Rb(11.13)4Bnr/J *rd3* mice and mislocalized to the inner segment of the In(5)30RK/J strain (Azadi et al., 2010; Cheng and Molday, 2013; Molday et al., 2013). GCAP1 and GCAP2 were detected at a reduced level in *rd3* mice and primarily confined to the inner segments as observed in GC1/GC2 knockout mice (Azadi et al., 2010; Karan et al., 2010). Other photoreceptor OS proteins including the cyclic GMP-gated channel, PDE6, peripherin-2, rhodopsin, and cone arrestin targeted normally to the OS of the *rd3* mice. Gene expression observed by microarray analysis indicated that the expression levels of genes encoding RD3, GC1 and GC2 in 21 day old (Rb(11.13)4Bnr *rd3* mice were similar to age-matched WT mice. Up-regulation of endothelin 2 (*EDn2*), glial fibrillary acid protein (*Gfap*), and complement component factor 1 (*Cf1*) and down-regulation of phosducin (*Pdc*), gap junction protein α5 (*Gja5*) and retinal G protein – coupled receptor (*Rgr*) genes, however, were observed for the *rd3* retina (Cheng and Molday, 2013).

The expression and distribution of GC1 was also examined in the In(5)30RK/J strain of *rd3* mice by immunofluorescence microscopy (Molday et al., 2013). GC1 was detected at reduced levels in this strain and confined primarily to the inner segments where it co-localized with the anti-KDEL ER marker. A small amount of GC1 was detected in the OS layer which may account for the attenuated scotopic and photopic ERGs observed in this strain. Similar mislocalization of GC in the *rcd2* collie has been observed. When the normal murine *Rd3* gene was delivered to photoreceptor cells of either strain of *rd3* mice using adeno-associated virus (AAV), GC1 and GC2 expressed at levels comparable to that of WT mice and localized normally to the photoreceptor OS layer (Molday et al., 2013). Collectively, these studies indicate that RD3 is required for efficient expression and localization of GCs to the photoreceptor OS.

The effect of RD3 expression on the localization of GC was also examined in transfected culture cells (Azadi et al., 2010). GC1 primarily localized to the ER of transfected cells in the absence of RD3, whereas RD3 co-localized with Rab11 in endosomes. Co-expression of GC1 and RD3 resulted in the exit of GC1 from the ER and colocalization with RD3 in Rab11 containing vesicles. These studies are consistent with the observed effect of RD3 in promoting the exit of GC from the ER in photoreceptor cells.

## RD3 INHIBITS GUANYLATE CYCLASE ACTIVITY

In addition to altering the expression level and trafficking of GC, RD3 inhibits its catalytic activity (Peshenko et al., 2011a). In the absence of GCAPs, RD3 expressed in either bacteria or HEK293 cells inhibited the basal and GCAP-activated GC catalytic activity at submicromolar concentrations, but did not alter the Ca^2+^ sensitivity of GCAPs. It remains to be determined if RD3 plays a significant role in modulating the activity of GCs during phototransduction. It is possible that RD3 only plays an important role in inhibiting cyclase activity during the trafficking of GCs within the inner segments. At present the quantity of RD3 in photoreceptors remains to be determined, but in *in vitro* measurements, RD3 at nanomolar concentrations competitively inhibited the activation of GC by GCAP (Peshenko et al., 2011a).

The effect of the RD3 mutation (F100ter) associated with LCA12 and other possible disease-linked missense mutations on the interaction of RD3 with GC was studied (Peshenko et al., 2011a). The F100ter and the G57V mutants were undetectable when expressed in HEK293 cells, but were expressed at significant levels in *E. Coli*. Other missense mutants (K130M, G35R, R68W, W6R/E23D) expressed at levels comparable to WT RD3 and bound GC1. The F100ter mutant had no effect of the cyclase activity, whereas the missense mutations inhibited GC activity to varying degrees. The W6R/E23D and G35R mutations inhibited the cyclase activity at a similar concentration as WT RD3, whereas the R68W, K130M and G57V were less effective. It remains to be determined if these missense mutations in RD3 alter the ability of RD3 to function in GC trafficking in photoreceptors.

## MODELS FOR THE ROLE OF RD3 IN PHOTORECEPTORS

**FIGURE 2:**
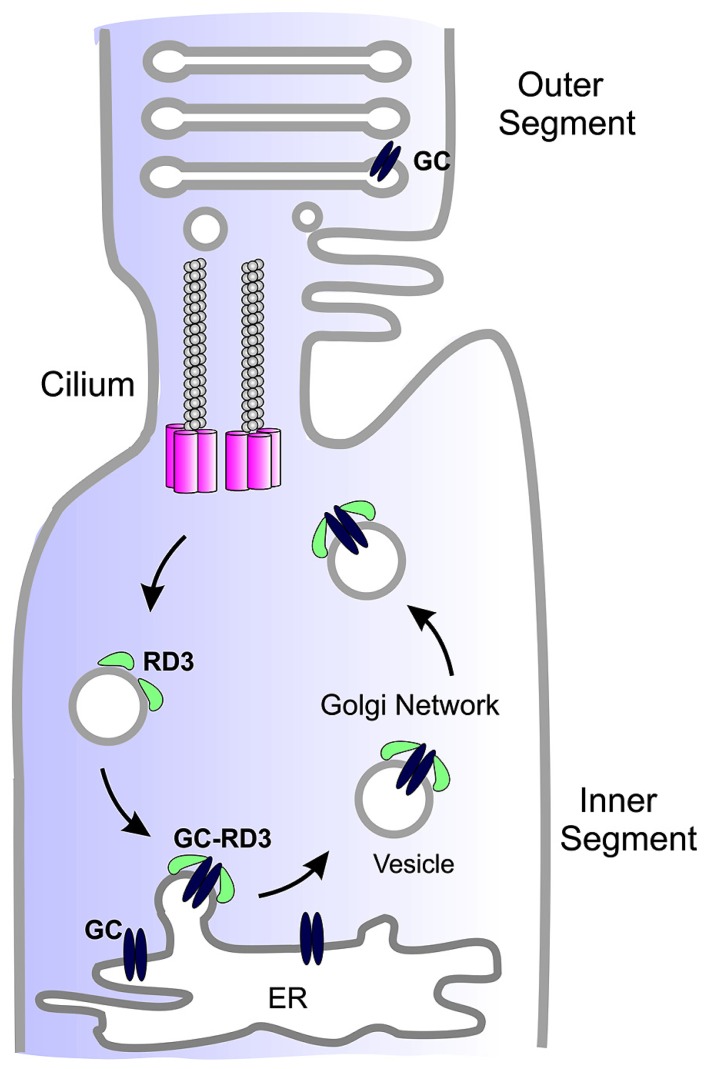
**Simplified diagram showing the role of RD3 in GC trafficking.** GC synthesized in the endoplasmic reticulum (ER) of photoreceptor inner segments binds to RD3 on the cytoplasmic side of the ER membrane. This interaction may induce membrane curvature required for the budding of GC-RD3 from the ER in the form of small vesicles. RD3 also inhibits the cyclase activity of GC. The RD3-GC complex together with other adaptor proteins is translocated through the Golgi to the base of the connecting cilium where GC perhaps in the absence of RD3 is transported through the cilium to the base of the outer segments where it is incorporated into disk membranes. At some point RD3 dissociates from GC possibly through phosphorylation or other post-translational modifications or protein–protein interaction. RD3 associated with endosomes is returned to the ER to initiate another cycle of RD3-mediated GC transport.

Studies carried out to date suggest that RD3 serves two roles in photoreceptor cells. A primary function of RD3 is to facilitate the exit of GC from the ER and its trafficking to the photoreceptor cilium (**Figure 2**). RD3 may also be involved in the trafficking of GC within the cilium although this remains to be determined.

A second function of RD3 is to inhibit GC catalytic activity (Peshenko et al., 2011a). The inhibition of GC activity by RD3 may be crucial for insuring that cGMP is not produced in the inner segment of photoreceptors cells during the trafficking of GC to the outer segment. In the absence of RD3 inhibition, GC could either use up the supply of GTP required for the function of other proteins including small G-proteins such as Rabs required for protein vesicle trafficking or overproduction of cGMP in the inner segment could be toxic to photoreceptors. In this regard it is interesting to note that an early study reported that retinal extracts from the *rcd2* collie showed a 10 times higher level of cGMP than control retinas during a 2–8 week period with only 25% reduction in PDE activity (Woodford et al., 1982).

## ROLE OF RD3 IN PHOTORECEPTOR CELL SURVIVAL

The mechanism by which loss in RD3 causes photoreceptor degeneration remains to be determined. Here we discuss two possible mechanisms. The first mechanism is centered on the role of GC and cGMP in maintaining calcium homeostasis in photoreceptors. It is generally known that too high or too low Ca^2+^ levels can be toxic to cells (Fain, 2006). Calcium homeostasis in OS is maintained by the influx of Ca^2+^ through the cGMP-gated channels and the efflux through the Na/Ca-K exchanger. In the absence of GC in the OS, cGMP will not be produced resulting in permanent closure of cGMP-gated channels and a reduction in intracellular Ca^2+^ below a threshold required for photoreceptor survival. In this case the mechanism of photoreceptor degeneration in the *rd3* mouse and LCA12 patients would be similar to photoreceptor degeneration in the GC1/GC2 knockout mouse and LCA1 patients. Direct comparison of the rate of photoreceptor degeneration in the *rd3* mouse and GC1/GC2 knockout mouse is complicated by the large variation in the rate of degeneration observed for different strains of *rd3* mice, although in general degeneration in the *rd3* mouse appears to be more rapid. Comparison of LCA12 with LCA1 patients is complicated by the presence of functional GC2 in rod photoreceptors of LCA1 patients. However, limited clinical assessment of these patients suggests that LCA12 is more severe (Preising et al., 2012; Perrault et al., 2013).

A second mechanism involves the role of RD3 in the inhibition of GC activity (Peshenko et al., 2011a). In the absence of RD3, GC in photoreceptor inner segments is likely to be active in catalyzing the production of cGMP (Woodford et al., 1982). Elevated cGMP levels can be toxic through the unregulated activation of cGMP-dependent enzymes. High cGMP levels have been implicated in photoreceptor cell death in the *rd1* and *rd10* mice linked to mutations in the β-subunit of PDE (Bowes et al., 1990; Chang et al., 2007), mice deficient in the A3 subunit of the cone cGMP-gated channel (Xu et al., 2013), and transgenic mice harboring an Y99C mutation in GCAP1 (Olshevskaya et al., 2004). Both the loss in RD3 chaperone activity and inhibition of GC activity may contribute to photoreceptor degeneration.

## Conflict of Interest Statement

The authors declare that the research was conducted in the absence of any commercial or financial relationships that could be construed as a potential conflict of interest.
